# Performance of a Norfentanyl Immunoassay in Specimens with Low Concentrations of Fentanyl and/or Norfentanyl

**DOI:** 10.1093/jalm/jfae036

**Published:** 2024-09-03

**Authors:** Sacha Uljon, Nicole V. Tolan, Grace K. Mahowald, Tahira Khaliq, Elizabeth D. Urwiller, Maria Daluz Fernandes, Sankha S. Basu, Phillip Kang, Timothy B. Erickson, Bryan D. Hayes, Peter R. Chai, Stacy E.F. Melanson

**Affiliations:** a Department of Pathology, Massachusetts General Hospital, Boston, MA, United States; b Department of Pathology, Harvard Medical School, Boston, MA, United States; c Department of Pathology, Brigham and Women’s Hospital, Boston, MA, United States; d Department of Emergency Medicine, Division of Medical Toxicology, Brigham and Women’s Hospital, Boston, MA, United States; e Department of Pharmacy, Massachusetts General Hospital, Boston, MA, United States.

## Abstract

**Background:**

Many fentanyl immunoassays are limited in their ability to detect norfentanyl. Urine specimens collected from individuals who have been exposed to fentanyl frequently have detectable concentrations of norfentanyl (≥2 ng/mL) but low concentrations of fentanyl (<2 ng/mL) by LC-MS/MS. The Lin-Zhi Fentanyl II Immunoassay (Lin-Zhi) claims 100% cross-reactivity with norfentanyl and therefore may detect exposure missed by other assays.

**Methods:**

In addition to verifying the manufacturer’s analytical sensitivity claims, we selected 92 urine specimens with low-positive Lin-Zhi results (1–99 absorbance units, lowest 10%) for analysis by the Immunalysis Health Equity Impact Assessment and ARK II fentanyl methods. The accuracy of the 3 immunoassays was compared to LC-MS/MS as the reference method.

**Results:**

Spiking studies using purified fentanyl and norfentanyl and a set of 100 consecutive specimens confirmed the manufacturer’s claims of limit of detection for fentanyl (3.8 ng/mL) and norfentanyl (5.0 ng/mL). However, the 92 low-positive patient specimens demonstrated concentrations of norfentanyl and fentanyl below 2.0 ng/mL by LC-MS/MS, with 47 (51%) having only norfentanyl detected. When comparing Lin-Zhi to the Immunalysis and ARK II immunoassays, only 27 (29%) of the 92 specimens were concordant. Fifty-two (57%) of the specimens were positive by LC-MS/MS and Lin-Zhi but false negative by one or both other immunoassays. Seven specimens (8%) were positive by Lin-Zhi but negative by the other immunoassays and had undetectable concentrations (<2 ng/mL) of fentanyl and norfentanyl by LC-MS/MS.

**Conclusions:**

The clinical sensitivity of the Lin-Zhi exceeds the manufacturer’s claims, providing results comparable to LC-MS/MS methods.

## BACKGROUND

Qualitative urine immunoassays are commonly used in clinical care to detect fentanyl exposure. Due to the limitations of immunoassays (i.e., false-negative or false-positive results), clinicians may request confirmatory testing by LC-MS/MS if a definitive result is necessary. However, confirmatory testing can be expensive and have longer turnaround times, limiting its utility. Choosing an immunoassay that is highly sensitive and specific improves clinical diagnosis in individuals while avoiding costly and time-consuming confirmatory testing.

Within the United States, there are currently 4 manufacturers of urine fentanyl immunoassays (Supplemental Table 1). A positive result is reported when the signal is above a predetermined threshold (e.g., “cut-off”). A higher signal typically corresponds to a higher concentration of fentanyl or metabolite in the urine specimen. This threshold is not typically reported to clinicians but can be used by laboratories to gauge the potential for false-positive results.

Urine immunoassays are primarily directed at the parent compound, fentanyl. However, less than 8% of fentanyl is excreted unchanged (1). Norfentanyl is the primary fentanyl metabolite and is present in urine at higher concentrations and for a longer duration than the parent drug. Approximately 30% of our LC-MS/MS specimens have norfentanyl concentrations of ≥2 ng/mL but <2 ng/mL for fentanyl. The first-generation ARK immunoassay (ARK I) claimed a 10% cross-reactivity to norfentanyl. However, an independent study reported a lower cross-reactivity of only 3% (2). The current generation, ARK II immunoassay, claims 7% cross-reactivity with norfentanyl, and this claim has borne out in independent studies (3, 4). The Thermo Fisher DRI II fentanyl immunoassay also claims 7% cross-reactivity with norfentanyl. The Lin-Zhi fentanyl II assay (Lin-Zhi), in contrast, claims 100% cross-reactivity with a 5 ng/mL cutoff for norfentanyl.

In this investigation, we sought to determine whether the reported improved cross-reactivity of the Lin-Zhi assay with norfentanyl would increase the ability to detect fentanyl exposure when compared to the Immunalysis and ARKII immunoassays. The hypothesis was that increased sensitivity would appear in specimens with norfentanyl concentrations at or near the cut-off and no detectable fentanyl.

## MATERIALS AND METHODS

Mass General Brigham Institutional Review Board approval was granted for this study (protocol number 2022P001640).

### Immunoassays

#### ARK II.

The qualitative automated ARK II Health Equity Impact Assessment (HEIA) (Ark Diagnostics) was performed at the Nantucket Cottage Hospital Laboratory on the Siemens Dimension EXL analyzer (Siemens Healthineers) following the manufacturer’s protocol. A manufacturer-supplied 1.0 fentanyl calibrator was used to define the HEIA assay cutoff. The enzymatic activity of the calibrator is normalized to a value of 1000, and signals of 1000 or more are considered positive.

#### Immunalysis.

The qualitative automated Immunalysis HEIA (Immunalysis Corporation) was performed on the Roche c502 analyzer (Roche Diagnostics) at Brigham and Women’s Hospital Laboratories following the manufacturer’s protocol. A manufacturer-supplied 4 ng/mL fentanyl calibrator was used to define the HEIA assay cutoff. The enzymatic activity of the calibrator was normalized to a value of 0, and signals greater than or equal to 0 were considered positive.

#### Lin-Zhi.

The Lin-Zhi qualitative automated HEIA (Lin-Zhi International) was performed on the Roche c502 analyzer (Roche Diagnostics) at Massachusetts General Hospital (MGH) following the manufacturer’s protocol. A manufacturer-supplied 5 ng/mL norfentanyl calibrator was used to determine the HEIA assay cutoff. The enzymatic activity of the calibrator was normalized to a value of 0, and signals greater than or equal to 0 were considered positive.

### LC-MS/MS

At Brigham and Women’s Hospital, fentanyl and norfentanyl were measured using a previously published dilute and shoot method developed for the determination of 38 prescription and illicit compounds in urine modified to incorporate a 2D liquid chromatography method (5). Briefly, 100 μL of spun urine is mixed with 700 μL of water and 200 μL of an internal standard working solution containing 100 ng/mL norfentanyl-D5 (Cerilliant). Using a Waters ACQUITY UPLC and Xevo TQ-S Micro Triple Quadrupole mass spectrometer, analysis was performed using a 2D trap and back-flush method, with positive electrospray ionization and multiple reaction monitoring for the qualitative determination of fentanyl and norfentanyl using a 3-point calibration curve at 1, 5, and 25 ng/mL. Ion transitions for fentanyl were m/z 337.2 → 188.2 and m/z 337.2 → 105.1 (qualifier ion). Ion transitions for norfentanyl were m/z 233.2 → 84.2 and m/z 233.2 → 55.6 (qualifier ion). The limit of detection for this assay is 1 ng/mL. This assay was used as the “gold standard” in all but the initial “all comers” Lin-Zhi validation.

At MGH, fentanyl and norfentanyl were measured in urine using the previously published method (6). An internal standard containing fentanyl-d5 and norfentanyl-d5 was added to each urine mixture to correct for volumetric and ionization variances in the LC/MS-MS. Aqueous calibrators were prepared to contain 0.0, 2.0, 20.0, and 200.0 μg/L fentanyl and 0.0, 8.0, 80.0, and 800.0 μg/L norfentanyl, respectively. To initiate the assay, 200 μL of each calibrator, control, and patient urine sample were mixed with 50 μL internal standard solution containing 80 μg/L fentanyl-d5 and norfentanyl-d5. Sixty μL of each mixture was injected into the LC-MS/MS. A Thermo Quantum Ultra triple quadrupole mass spectrometer (Thermo Fisher Scientific) and TLX UPLC system (Thermo Fisher Scientific) equipped with a heated electrospray interface was operated in the positive ion mode using online extraction. The following transitions were monitored by LC-MS/MS: 337 m/z (precursor ion) 188 m/z (quantifier ion), and 105 m/z (qualifier ion) for fentanyl; 342 m/z (precursor ion) → 188.2 m/z (quantifier ion) for fentanyl-d5; 233 m/z (precursor ion) → 84 m/z (quantifier ion) and 150 m/z (qualifier ion) for norfentanyl; and 238 m/z (precursor ion) → 84 m/z (quantifier ion) for norfentanyl-d5. In this quantitative assay, the limit of quantitation and detection are the same for fentanyl and norfentanyl (2 ng/mL). This assay was used only in the “all comers” validation.

### Spiking Studies

**Fig. 1.**
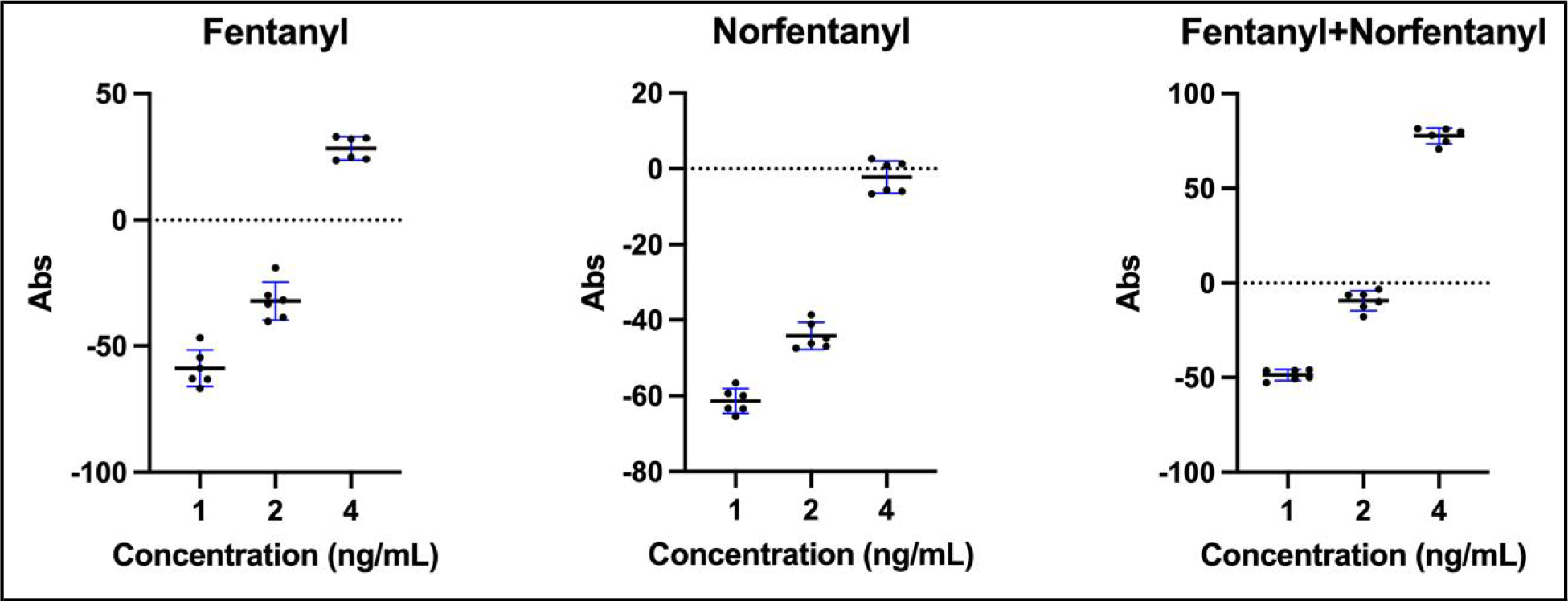
Spiking study results for fentanyl (left), norfentanyl (middle), or equal concentrations of both (right). Individual measurements are represented by black dots, the line shows the mean of six measurements, and the SD is represented by the blue error bars. Absorbance (Abs) is in arbitrary units, with 0 being the positive threshold.

Fentanyl, norfentanyl, and deuterated internal standards were purchased from Cerilliant. A stock concentration of 1 mg/mL was made in methanol. Serial dilutions of fentanyl, norfentanyl, or both in equal concentrations were made in water to produce the concentrations in Fig. 1 (4 ng/mL, 2 ng/mL, 1 ng/mL, 0.5 ng/mL). Each sample was run 6 times on the Lin-Zhi immunoassay.

### Specimen Selection

#### Lin-Zhi Immunoassay Verification.

As part of the initial assay verification studies, 100 consecutive urine specimens submitted for the Lin-Zhi fentanyl immunoassay across all clinical care areas were collected and run on the LC-MS/MS at MGH using the method described.

#### Low-Positive Set.

Ninety-two residual waste specimens with low-positive Lin-Zhi were retrieved for additional testing. The specimens were split into aliquots to minimize freeze-thaw cycles. Each specimen underwent a total of 2 freeze-thaw cycles. One aliquot was sent to Brigham and Women’s Hospital for LC-MS/MS and Immunalysis testing, and a second aliquot was sent to Nantucket Cottage Hospital and run on the ARK II.

## RESULTS

### Spiking Studies

Spiking studies were performed to confirm the sensitivity claims of the manufacturer for the Lin-Zhi assay for fentanyl (3.8 ng/mL) and norfentanyl (5.0 ng/mL) (Fig. 1). We used the same solutions that we use to calibrate the LC-MS/MS method. One hundred percent of fentanyl-only specimens were positive at 4.0 ng/mL, consistent with the manufacturers claims of 3.8 ng/mL (Fig. 1, left). The sensitivity for norfentanyl was improved compared to the package insert claim of 5.0 ng/mL; approximately 50% of samples at 4 ng/mL (Fig. 1, middle) were positive. As expected, specimens spiked with both norfentanyl and fentanyl required lower concentrations of each as the signal is additive (Fig. 1, right).

### Lin-Zhi Immunoassay Verification Results

The concordance of the Lin-Zhi assay with LC-MS/MS was 100% in 100 consecutive urines (16 positive and 84 negative). Interestingly, some specimens with measurable fentanyl and/or norfentanyl concentrations (>2 ng/mL) by LC-MS/MS but below the manufacturer’s reported cross-reactivity claims gave positive results.

### Low-Positive Specimen Results

**Fig. 2.**
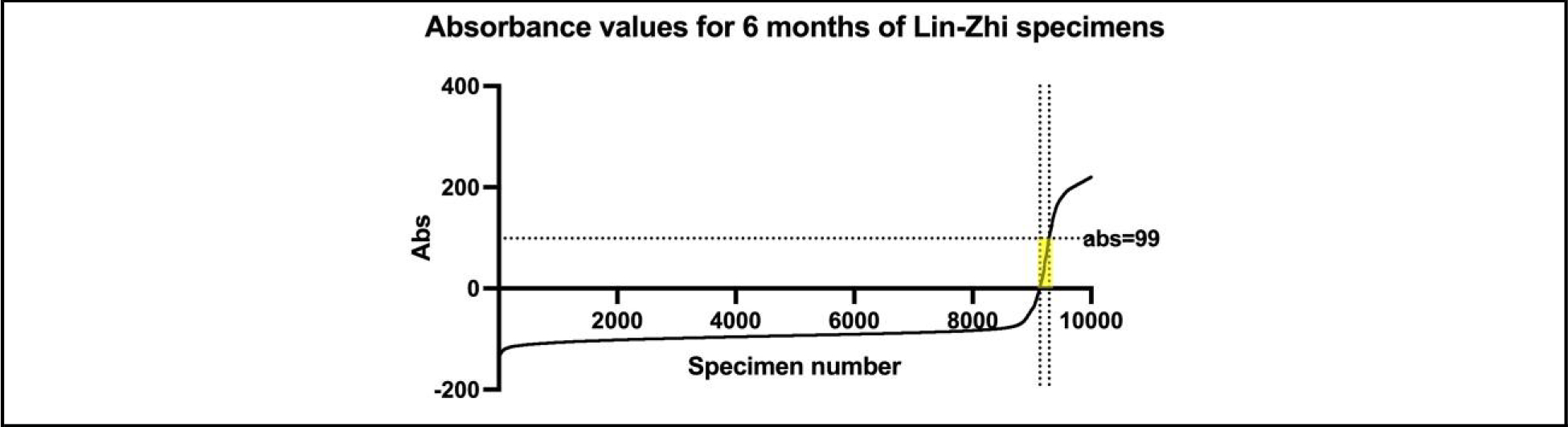
Absorbance values from the first 10 000 of the Lin-Zhi immunoassay specimens are displayed. The area chosen for the study (Abs 1–99) is highlighted with the horizontal dotted line indicating the value to 99. The x axis shows specimen number, ranked by Abs value. Abs, absorbance, arbitrary units, where >0 is reported as positive.

A total of 10 774 specimens were tested at MGH during the first 6 months of implementing the Lin-Zhi method, of which 1331 (13%) were positive. Of these, 124 (9%) had absorbances between 0 and 99 (Fig. 2). A total of 92 (74%) residual waste specimens were retrieved for LC-MS/MS and Immunalysis fentanyl immunoassay testing where 78 (63%) had sufficient volume to also perform the ARK II fentanyl assay (Supplemental Table 2).

A total of 47 (51%) of the 92 low-positive specimens had detectable concentrations of norfentanyl by LC-MS/MS but undetectable concentrations of fentanyl (<1 ng/mL), (Table 1), only 6 of which were positive by all 3 immunoassays.

**Fig. 3.**
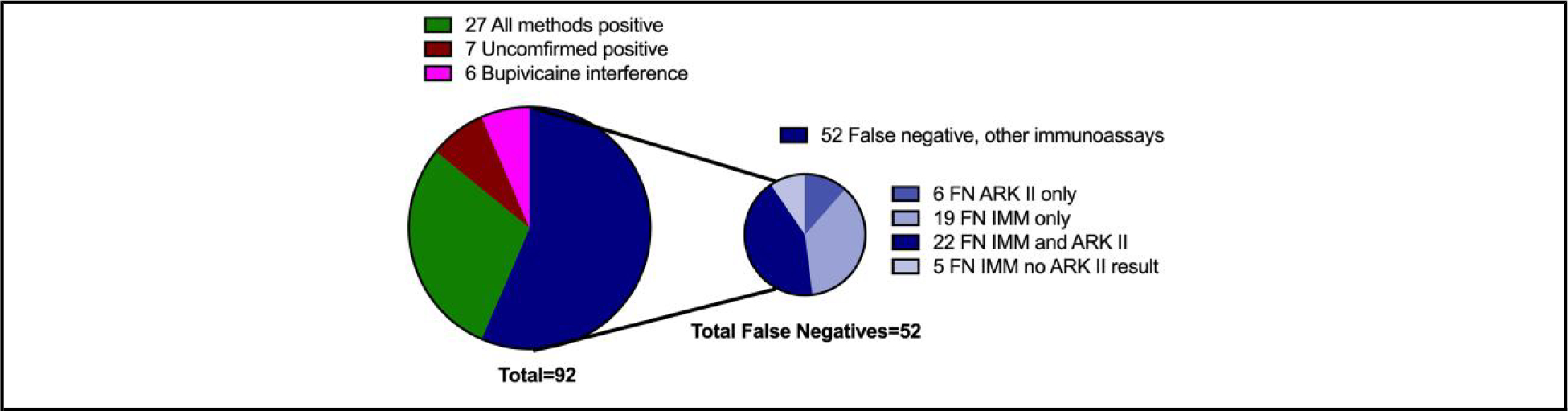
Pie chart showing result categories for the 92 low positive specimens. As shown in the smaller pie chart, 52 were categorized as FN by either IMM (light blue), ARK II [ARK (medium blue)], or both (dark blue). Twenty-seven were positive by all methods performed (green); 6 had bupivacaine interference and LC-MS/MS results could not be reported (pink); and 7 were positive by Lin-Zhi, negative by IMM and ARK but had undetectable fentanyl and norfentanyl by LC-MS/MS (i.e., unconfirmed positives) (dark red). FN, false negative; IMM, immunalysis.

Only 27 of the 92 specimens (29%) were concordant by LC-MS/MS, Lin-Zhi, Immunalysis, and ARK II (if performed) (Fig. 3 and Supplemental Table 2). The majority 52 (57%) were positive by LC-MS/MS and Lin-Zhi but falsely negative by Immunalysis assay, ARK II assay, or both (Fig. 3). For 6 (7%) of the 92 specimens, norfentanyl could not be reported due to bupivacaine interference on LC-MS/MS (6) (Fig. 3, Supplemental Table 2). Three of these cases were confirmed to be from patients who had received both bupivacaine and fentanyl. In the remaining 3 specimens, collected from neonates, we verified administration of a fentanyl/bupivacaine epidural to the mother during labor, which can be detected in neonatal urine (7, 8).

Seven of the 92 (8%) specimens were classified as unconfirmed positives because they had undetectable concentrations (<1 ng/mL) of both fentanyl and norfentanyl on LC-MS/MS but were positive by one or more immunoassays (Table 2). Specimens F1 and F2 were from patients with known dextromethorphan exposure, in one case as a suicide attempt. According to the manufacturer, high concentrations of dextromethorphan (40 000 ng/mL) can cause false-positive Lin-Zhi results. We suspect that, given the clinical context, the positive Lin-Zhi result was likely a false positive in the F1 and F2 samples. Specimens F3 and F4 were from a patient who was brought to the emergency department after she was found down with altered mental status. The patient reported ingesting edibles with unknown contents. Urine screens for amphetamines, benzodiazepines, cocaine metabolite, opioids, and oxycodone were negative. Specimen F5 was from a woman with no reported history of fentanyl use. Specimen F6 was from an individual with a history of substance use who also tested positive for benzodiazepines, cocaine, and buprenorphine. Finally, specimen F7 was from a patient who was given one dose of fentanyl (50 micrograms) 8 hours prior to specimen collection.

## DISCUSSION

### Significance

Increasing adulteration of illicit drugs with fentanyl and fentanyl use in individuals with opioid use disorder necessitates a reliable and rapid urine immunoassay screen. There are multiple FDA-cleared commercially available immunoassays. However, we demonstrate that the Lin-Zhi assay is more sensitive than other immunoassays due to its higher cross-reactivity with norfentanyl, particularly when norfentanyl is present in the absence of the parent fentanyl compound. Given the increased sensitivity, the adoption of the Lin-Zhi assay, or future assays with high cross-reactivity to norfentanyl, poses important considerations for clinical laboratories and toxicology services.

Use of the Lin-Zhi assay is likely to result in more positive results. Individuals who were exposed to fentanyl, whether intentionally or unintentionally, may have positive urine tests for longer periods of time, since norfentanyl is present in urine for longer than fentanyl in most individuals. In fact, some heavy users continue to test positive for norfentanyl by LC-MS/MS many days after use (9); these would previously have been missed by immunoassay. Additionally, higher norfentanyl:fentanyl ratios have been reported in pregnant women with a history of fentanyl use, suggesting potentially altered metabolism in pregnancy (10, 11). Therefore, an assay that is more sensitive to norfentanyl may disproportionately affect these vulnerable groups and lead to false assumptions regarding timing of use. We have had obstetrical and transplant patients who were flagged for a positive immunoassay, but the levels were so low by LC-MS/MS that the clinical teams eventually concluded that the use was likely to be remote. Unfortunately, there is no way to know this for sure.

In contrast, the increased sensitivity has implications for the management of important populations such as those with opioid use disorder on therapy. Initiation of buprenorphine therapy may lead to high concentrations of opioids in circulation and precipitate withdrawal. Practitioners should be made aware that a positive immunoassay in these patients can be triggered by an inactive metabolite in the urine and therefore may not be clinically concerning.

The high sensitivity of the Lin-Zhi may also pose some conundrums when comparing immunoassay results to confirmatory LC-MS/MS methods. In this cohort, despite using a LC-MS/MS method with a relatively low limit of detection (1 ng/mL), 7 specimens positive by Lin-Zhi were unconfirmed by LC-MS/MS. Many of these specimens had detectable concentrations of fentanyl and/or norfentanyl by LC-MS/MS; however, the concentrations were less than 1 ng/mL and were therefore reported as negative. One unconfirmed positive (F7, Table 2) received one injection of Sublimize 8 hours earlier, suggesting the Lin-Zhi result was a true positive. Further results from 6 specimens were unable to be reported by LC-MS/MS due to bupivacaine interference. These specimens were from patients who were administered fentanyl as part of their medical care, suggesting these cases were true positives on Lin-Zhi. The improved sensitivity of the Lin-Zhi when compared to LC-MS/MS may be due to the cut-off of 1 ng/mL utilized in our laboratory or to cross-reactivity with other, minor fentanyl metabolites that are not detected on our targeted LC-MS/MS methods.

Given the improved sensitivity of the Lin-Zhi method, it may have decreased specificity, leading to a higher false-positive rate. Other assays that claim significant cross-reactivity with norfentanyl include the ARK I, ARK II, and 2 generations of the Thermo Fisher DRI assay. The first generations of both immunoassays have high false-positive rates (3, 12). Of the 92 low positives on the Lin-Zhi, 7 specimens did not confirm by LC-MS/MS. F1 and F2 were from patients who reported dextromethorphan use, which is listed in the package insert as a potential cause of false positives. We were unable to determine the true result in 4 specimens (F3 to F6). As stated earlier, F7 was a true-positive result. In this study, the false-positive rate could be as high as 6/92 or 6.5%. However, given that these specimens were deliberately chosen to enrich for false positives, the false-positive rate in the general population is likely to be lower. Indeed, there were no false positives in our validation set of 100 consecutive specimens.

### Limitations

Our study has limitations. We focused on specimens with low-positive absorbance (bottom 10%) values by the Lin-Zhi assay to interrogate the sensitivity limits of the assay and therefore cannot determine the sensitivity and specificity of the assay in the general population. Further, because the LC-MS/MS and all 3 immunoassays are all calibrated differently and use different quality control materials, direct concentrations are difficult to compare on orthogonal methods.

Our set of consecutive specimens (100) is too small to detect a false-positive rate with certainty. However, a recent unbiased study of 250 specimens reported a sensitivity and specificity of 98% and 96.8%, respectively (13).

We did not conclusively determine the cause of 6 of the 7 unconfirmed positives. While 2 of the 6 are likely false positives due to dextromethorphan abuse, we did not confirm this by measuring the concentration of dextromethorphan in the specimens. Likewise, we did not confirm if fentanyl analogues (seen in edible gummies) or a mixture of metabolites were present in the remaining specimens that would explain the results.

## CONCLUSION

The Lin-Zhi qualitative rapid immunoassay can be utilized in multiple clinical settings including emergency departments, medical toxicology services, and behavioral health addiction treatment centers. In our study, the sensitivity of the Lin-Zhi assay for detecting fentanyl exposure met or exceeded the manufacturer’s claims. Laboratories that use the Lin-Zhi will detect cases where <1 ng/mL of fentanyl is present. This may or may not be desirable depending on the clinical context. The testing characteristics of the Lin-Zhi assay should be taken into consideration when interpreting the test results, especially in cases where recent use has different clinical or medical/legal implications compared to distant use.

## Supplementary Material

supplementary material

## Figures and Tables

**Table 1.** Specimens positive for NFENT and negative for FENT.

Number	LC-MS/MS		Immunoassay		
	FENT (ng/mL)	NFENT (ng/mL)	Lin-Zhi	IMM	ARK II
N1	<1	1.0	POS	NEG	NEG
N2	<1	1.1	POS	NEG	POS
N3	<1	1.2	POS	NEG	NEG
N4	<1	1.4	POS	NEG	NEG
N5	<1	1.5	POS	POS	POS
N6	<1	1.6	POS	NEG	NEG
N7	<1	1.8	POS	NEG	NEG
N8	<1	1.8	POS	NEG	NEG
N9	<1	2.0	POS	POS	NEG
N10	<1	2.0	POS	NEG	NEG
N11	<1	2.1	POS	NEG	NEG
N12	<1	2.2	POS	NEG	NEG
N13	<1	2.2	POS	NEG	NEG
N14	<1	2.2	POS	NEG	N/A
N15	<1	2.3	POS	POS	N/A
N16	<1	2.3	POS	NEG	NEG
N17	<1	2.3	POS	NEG	NEG
N18	<1	2.4	POS	NEG	N/A
N19	<1	2.5	POS	NEG	N/A
N20	<1	2.6	POS	NEG	N/A
N21	<1	2.7	POS	POS	NEG
N22	<1	2.9	POS	NEG	POS
N23	<1	3.0	POS	POS	POS
N24	<1	3.3	POS	NEG	NEG
N25	<1	3.5	POS	NEG	NEG
N26	<1	3.5	POS	NEG	NEG
N27	<1	3.6	POS	NEG	POS
N28	<1	3.7	POS	POS	POS
N29	<1	3.8	POS	POS	POS
N30	<1	4.1	POS	NEG	NEG
N31	<1	4.4	POS	NEG	NEG
N32	<1	4.6	POS	NEG	NEG
N33	<1	4.7	POS	NEG	NEG
N34	<1	5.5	POS	NEG	POS
N35	<1	6.0	POS	POS	NEG
N36	<1	6.5	POS	NEG	NEG
N37	<1	7.1	POS	NEG	POS
N38	<1	7.2	POS	POS	NEG
N39	<1	8.0	POS	POS	POS
N40	<1	8.0	POS	POS	POS
N41	<1	8.1	POS	POS	NEG
N42	<1	8.3	POS	NEG	POS
N43	<1	8.3	POS	POS	POS
N44	<1	9.6	POS	NEG	POS
N45	<1	9.7	POS	POS	POS
N46	<1	9.7	POS	NEG	POS
N47	<1	9.9	POS	NEG	POS

NFENT, norfentanyl; FENT, fentanyl; IMM, immunalysis; POS, positive; NEG, negative; N/A, not applicable (the assay was not performed).

Values under 1 ng/mL are interpreted as negative in the LC-MS/MS assay.

**Table 2.** Results of unconfirmed positive specimens.

Number	LC-MS/MS Data			Immunoassay data			Interpretation
	FENT (ng/mL)	NFENT (ng/mL)	Interp	Lin-Zhi	IMM	ARK II	
F1	<1	<1	NEG	POS	NEG	NEG	FP Lin-Zhi likely due to dextromethorphan
F2	<1	<1	NEG	POS	NEG	NEG	FP Lin-Zhi likely due to dextromethorphan
F3	<1	<1	NEG	POS	NEG	N/A	Edible gummy, unexplained FP Lin-Zhi
F4	<1	<1	NEG	POS	NEG	N.A	Edible gummy, unexplained FP Lin-Zhi
F5	<1	<1	NEG	POS	NEG	N/A	No documented ingestions, unexplained FP Lin-Zhi
F6	<1	<1	NEG	POS	POS	NEG	Many unprescribed substances, unexplained FP Lin-Zih and IMM
F7	<1	<1	NEG	POS	NEG	NEG	Received fentanyl (SUMBLIMAZE IV) 8 h prior, true-positive Lin-Zih

FENT, fentanyl; NFENT, norfentanyl; IMM, immunalysis; POS, positive; NEG, negative; FP, false positive; N/A, not applicable (the assay was not done). Values under 1 ng/mL are interpreted (Interp) as negative in the LC-MS/MS assay.

## IMPACT STATEMENT

In this manuscript, we compare 3 different urine fentanyl immunoassays with mass spectrometry. We present evidence that a urine fentanyl immunoassay (Lin-Zhi brand) can detect amounts of norfentanyl that may reflect remote exposure rather than recent use. It will benefit patients in recovery from substance use disorder who may metabolize fentanyl differently, leading to lingering positivity on urine screens.

## Nonstandard Abbreviations:

IA: immunoassay
HEIA: homogeneous enzyme immunoassay
