# Supplementary material for: Performance of a Norfentanyl Immunoassay in Specimens with Low Concentrations of Fentanyl and/or Norfentanyl
Source: J Appl Lab Med. Author manuscript; Available in PMC 2024 Sep 4. (PMC11371532; doi:10.1093/jalm/jfae036)
Supplement: supplementary material [file NIHMS1991955-supplement-supplementary_material.docx]

**Supplemental Material**

**Supplemental Table 1**. Urine fentanyl immunoassays available in the US and their performance claims.

| **Assay manufacturer and name** | **Cut-off**  **(ng/mL)** | **Norfentanyl cross-reactivity** | **Concentration of Norfentanyl Triggering a Positive Result (ng/mL)** |
| --- | --- | --- | --- |
| *Lin-Zhi - Fentanyl II Enzyme Immunoassay | 5.0** | 100% | 5.0 |
| ARK Diagnostics - ARK^TM^ Fentanyl Assay | 1.0 | 10% | 10.0 |
| *ARK Diagnostics - ARK^TM^ Fentanyl II Assay | 1.0 | 7% | 15.0 |
| Thermo Fisher Scientific - DRI^TM^ Fentanyl II Assay | 1.0 | 7% | 15.0 |
| *Immunalysis - Fentanyl Urine Enzyme Immunoassay (HEIA) | 4.0 | <1% | 100,000 |
| Immunalysis - Fentanyl Urine Enzyme Immunoassay (SEFRIA) | 1.0 | <1% | 20,000 |

*Evaluated in this study

**Calibrated with norfentanyl instead of fentanyl. The fentanyl cut-off is 3.8 ng/mL.

HEIA = Homogenous enzyme immunoassay, SEFRIA = Synthetic enzyme fragment immunoassay

**Supplemental Table 2. Results of 92 Low Positive Specimens**

|  | **LC-MS/MS** | | **Immunoassay** | | |  |
| --- | --- | --- | --- | --- | --- | --- |
| **Label** | **FEN**  **(ng/mL)** | **NFEN (ng/mL)** | **Lin-Zhi** | **IMM** | **ARKII** | **Interpretation** |
|  | 6.1 | 1.1 | POS | POS | POS | All positive |
| N45 | <1 | 9.7 | POS | POS | POS | All positive |
| N43 | <1 | 8.3 | POS | POS | POS | All positive |
|  | 4.2 | 5.2 | POS | POS | POS | All positive |
| N39 | <1 | 8.0 | POS | POS | POS | All positive |
|  | 2.3 | 2.0 | POS | POS | POS | All positive |
|  | 10.8 | 1.8 | POS | POS | POS | All positive |
| N29 | <1 | 3.8 | POS | POS | POS | All positive |
|  | 9.3 | <1 | POS | POS | POS | All positive |
|  | 1.5 | 2.9 | POS | POS | POS | All positive |
|  | 2.9 | 2.9 | POS | POS | POS | All positive |
|  | 2.9 | 2.2 | POS | POS | POS | All positive |
| N5 | <1 | 1.5 | POS | POS | POS | All positive |
| N28 | <1 | 3.7 | POS | POS | POS | All positive |
|  | 2.1 | 4.7 | POS | POS | POS | All positive |
|  | 7.2 | <1 | POS | POS | POS | All positive |
| N23 | <1 | 3.0 | POS | POS | POS | All positive |
| N40 | <1 | 8.0 | POS | POS | POS | All positive |
|  | 5.0 | <1 | POS | POS | POS | All positive |
|  | 1.5 | 8.7 | POS | POS | POS | All positive |
|  | 2.0 | 8.3 | POS | POS | POS | All positive |
|  | 2.9 | <1 | POS | POS | POS | All positive |
|  | 6.1 | <1 | POS | POS | N/A | All positive, no ARKII |
|  | 1.5 | <1 | POS | POS | N/A | All positive, no ARKII |
|  | 3.7 | 1.9 | POS | POS | N/A | All positive, no ARKII |
|  | 1.5 | 4.4 | POS | POS | N/A | All positive, no ARKII |
| N15 | <1 | 2.3 | POS | POS | N/A | All positive, no ARKII |
|  | <1 | UTR | POS | NEG | N/A | bupivicaine positive |
|  | <1 | UTR | POS | NEG | POS | bupivicaine positive |
|  | <1 | UTR | POS | NEG | POS | bupivicaine positive |
|  | 1.0 | UTR | POS | NEG | NEG | bupivicaine positive |
|  | <1 | UTR | POS | NEG | NEG | bupivicaine positive |
|  | <1 | UTR | POS | POS | POS | bupivicaine positive |
| N35 | <1 | 6.0 | POS | POS | NEG | FN ARKII |
| N38 | <1 | 7.2 | POS | POS | NEG | FN ARKII |
| N9 | <1 | 2.0 | POS | POS | NEG | FN ARKII |
| N21 | <1 | 2.7 | POS | POS | NEG | FN ARKII |
| N41 | <1 | 8.1 | POS | POS | NEG | FN ARKII |
|  | 1.2 | 8.0 | POS | POS | NEG | FN ARKII |
| N47 | <1 | 9.9 | POS | NEG | POS | FN IMM |
| N42 | <1 | 8.3 | POS | NEG | POS | FN IMM |
|  | 1.2 | 4.5 | POS | NEG | POS | FN IMM |
|  | 1.9 | 0.5 | POS | NEG | POS | FN IMM |
|  | 1.0 | 1.6 | POS | NEG | POS | FN IMM |
| N27 | <1 | 3.6 | POS | NEG | POS | FN IMM |
|  | 1.3 | 13.1 | POS | NEG | POS | FN IMM |
|  | 1.2 | 1.3 | POS | NEG | POS | FN IMM |
| N37 | <1 | 7.1 | POS | NEG | POS | FN IMM |
|  | 1.0 | 6.2 | POS | NEG | POS | FN IMM |
| N2 | <1 | 1.1 | POS | NEG | POS | FN IMM |
|  | 1.1 | 3.1 | POS | NEG | POS | FN IMM |
|  | 1.2 | 2.2 | POS | NEG | POS | FN IMM |
| N46 | <1 | 9.7 | POS | NEG | POS | FN IMM |
| N34 | <1 | 5.5 | POS | NEG | POS | FN IMM |
| N22 | <1 | 2.9 | POS | NEG | POS | FN IMM |
|  | 1.1 | 1.7 | POS | NEG | POS | FN IMM |
| N44 | <1 | 9.6 | POS | NEG | POS | FN IMM |
|  | 2.4 | 4.8 | POS | NEG | POS | FN IMM |
| N18 | <1 | 2.4 | POS | NEG | N/A | FN IMM, no ARKII |
| N20 | <1 | 2.6 | POS | NEG | N/A | FN IMM, no ARKII |
|  | 1.2 | 6.2 | POS | NEG | N/A | FN IMM, no ARKII |
| N19 | <1 | 2.5 | POS | NEG | N/A | FN IMM, no ARKII |
| N14 | <1 | 2.2 | POS | NEG | N/A | FN IMM, no ARKII |
| N25 | <1 | 3.5 | POS | NEG | NEG | FN IMM and ARKII |
| N11 | <1 | 2.1 | POS | NEG | NEG | FN IMM and ARKII |
| N32 | <1 | 4.6 | POS | NEG | NEG | FN IMM and ARKII |
| N31 | <1 | 4.4 | POS | NEG | NEG | FN IMM and ARKII |
| N16 | <1 | 2.3 | POS | NEG | NEG | FN IMM and ARKII |
| N7 | <1 | 1.8 | POS | NEG | NEG | FN IMM and ARKII |
| N4 | <1 | 1.4 | POS | NEG | NEG | FN IMM and ARKII |
| N36 | <1 | 6.5 | POS | NEG | NEG | FN IMM and ARKII |
| N26 | <1 | 3.5 | POS | NEG | NEG | FN IMM and ARKII |
| N24 | <1 | 3.3 | POS | NEG | NEG | FN IMM and ARKII |
| N6 | <1 | 1.6 | POS | NEG | NEG | FN IMM and ARKII |
| N33 | <1 | 4.7 | POS | NEG | NEG | FN IMM and ARKII |
| N12 | <1 | 2.2 | POS | NEG | NEG | FN IMM and ARKII |
| N10 | <1 | 2.0 | POS | NEG | NEG | FN IMM and ARKII |
|  | 1.2 | 1.3 | POS | NEG | NEG | FN IMM and ARKII |
| N13 | <1 | 2.2 | POS | NEG | NEG | FN IMM and ARKII |
| N8 | <1 | 1.8 | POS | NEG | NEG | FN IMM and ARKII |
| N3 | <1 | 1.2 | POS | NEG | NEG | FN IMM and ARKII |
| N17 | <1 | 2.3 | POS | NEG | NEG | FN IMM and ARKII |
| N1 | <1 | 1.0 | POS | NEG | NEG | FN IMM and ARKII |
|  | 1.3 | 2.5 | POS | NEG | NEG | FN IMM and ARKII |
| N30 | <1 | 4.1 | POS | NEG | NEG | FN IMM and ARKII |
| F1 | <1 | <1 | POS | NEG | NEG | UCF Lin-Zhi |
| F2 | <1 | <1 | POS | NEG | NEG | UCF Lin-Zhi |
| F4 | <1 | <1 | POS | NEG | N/A | UCF Lin-Zhi |
| F3 | <1 | <1 | POS | NEG | N/A | UCF Lin-Zhi |
| F5 | <1 | <1 | POS | NEG | N/A | UCF Lin-Zhi |
| F7 | <1 | <1 | POS | NEG | NEG | UCF Lin-Zhi |
| F6 | <1 | <1 | POS | POS | NEG | UCF Lin-Zhi and IMM |

LC-MS/MS = liquid chromatography-tandem mass spectrometry, FENT = fentanyl, NFENT = norfentanyl, Lin-Zhi = Lin-Zhi fentanyl II immunoassay, IMM = Immunalysis immunoassay, ARKII = ARK II immunoassay, NEG = negative, POS = positive, FN = false negative, UCF = unconfirmed

Column 1 is the identity of the specimen also appears in Table I or Table II. Columns 2 and 3 are the LC-MS/MS results, where <1ng/mL is considered negative and 1 or more ng/mL is positive. Bupivacaine interference indicates that norfentanyl could not be reported. Columns 4-6 are the qualitative immunoassay results. Column 7 is the description of how the assays compare.
